# New Theory of the Influence of Variety in Diet in Health and Disease

**Published:** 1833-01-01

**Authors:** 


### VIII.

### New Theory of the Influence of Variety in Diet, in Health and Disease, &c. &c. By *Charles Cameron*, Surgeon, R.N. 8vo. pp. 104. Highley. Oct. 1832.

We like the motto which Mr. Cameron has placed on the title-page of his book,—" Nemo me impune lacessit." There is something as refreshing in it as in the air on the brow of Ben Cruachan—something as bold as the offer of Evan Dhu, when his chief was condemned to death at Carlisle, to clear the Court with his single claymore, if the judge would only give him that chance, and sit by to see fair play. We suspect that the faithful Maccombich would have been more successful in putting the Saxons of Carlisle to the rout, with his trusty claymore, than will his countryman, Mr. Cameron, in establishing his " New Theory," in opposition to the critics of the day.

Our author appears to proceed on the fundamental principle that man cannot be nourished completely by any one species of food, however rich in quality, unless that food can yield " all the elementary principles which enter into the formation of the human frame."

" The only article of diet which nature has provided for the use of man, capable of fully accomplishing this object, is milk. Milk alone, and no other aliment, contains all the elementary principles required for the restoration of waste, nourishment, and health of the human body ; and the writer wishes *this* to be particularly borne in mind by the reader while perusing the following essay." viii.

But as all men cannot get, or may not like milk alone, for diet, the next principle is to *vary* the diet, that all the elementary constituents of the living machine may be therein found. Dr. Paris and others, indeed, maintain that,—

" As every description of food, whether derived from the animal or vegetable kingdom *is converted into blood,* it may be inferred that THE ULTIMATE EFFECT OF ALL ALIMENTS MUST BE VIRTUALLY THE SAME ; and that the *several species can only differ from each other* IN THE QUANTITY OF NUTRIMENT THEY AFFORD, in the comparative degree of stimulus they impart to the organs through which they pass, and in the proportion of vital energy they require for their assimilation.' (See ' Paris on Diet,' paragraph 114), and (in paragraph 61) he says, that after the lapse of 24 hours—' the food has been decomposed, and recombined into compounds analogous to those which compose the organs to which it is carried ; and *this appears to take place with the same facility,* HOWEVER REMOTE THE COMPOSITION OF THE ALIMENT MAY BE FROM THAT OF THE SUBSTANCES WITH WHICH IT IS ASSIMILATED.' " 9.

This doctrine, says Mr. Cameron, is not that of an individual, but of the whole profession throughout the civilized world. " It is a doctrine which has led man away from the truth for ages, and has exerted the most baneful influence over the practice of physic." After much good and specious reasoning on the superiority of milk and vegetables over other kinds of food, the author has recourse to a dangerous kind of support—prophecy.

" I have thus endeavoured to shew that there is one aliment provided by nature, and but one, which is alone capable of supporting human life without any other food. And although milk was evidently intended by Providence as the sole nutriment during the early period of our lives, and for this period only, it would, in every probability, support life at any period, with few other additional elementary substances—we shall say bread and vegetables—far better than any other diet consisting of so limited a variety. It will also be found that those persons who use much milk as food are not subject to epidemic putrid diseases, in nearly the same proportion, as those who live in villages and towns, and use but little milk as an article of diet. In the country parts of Scotland, where people live chiefly on different preparations of oatmeal and milk, *I would venture to foretel with much confidence, that they will suffer but very little from the present prevailing epidemic cholera, or any other malignant or putrid disease, because these two aliments contain nearly all the elements entering into the formation of our bodies ; and this, with some vegetable matter or fruit, and some table salt, would probably support life and health, better than any other diet which nature has provided for our sustenance, consisting of so small a variety of articles.*" 35.

The foregoing passage demonstrates that Mr. Cameron's book has been long in the press, and proves also, that he is an inexperienced author, otherwise he would have cancelled the above prediction, even at some expence, and not exposed himself to the jeers of some of those cynical critics who will not be slow to pluck the thistle, notwithstanding the threatening motto of "Nemo me impune lacessit." Although we are far from agreeing with the vulgar prejudice that cholera is produced by cabbage, or even by kail, yet we are much mistaken if the diet of the Scotch, like that of our Gallic neighbours, has not aggravated the mortality of a disease which attacks the digestive organs so fiercely. There may be more safety in the following passage.

"And secondly, I have attempted to shew that the general tendency of a continued course of any food, which is deficient in the variety of elementary substances requisite for repairing the waste of the body, is invariably to produce a diseased condition of the fluids and solids, and which may exist for a long period without attracting attention, but, if the cause be continued, will eventually end in that disease known as scurvy. That this condition, although noticed only by the initiated, is of very common occurrence in all our large towns, and is easily observed in the complexions of individuals while walking the streets. I would say, that at least one-tenth of the population are thus affected to a considerable degree, and one-third more or less so. If they be examined while labouring under the influence of this condition of the body, it will be found that their complexion is of a dark, tawny, unhealthy hue. Their gums will be found swelled and tender, and their tongue of a purple shade, which is quite characteristic, although not conspicuous, in the earlier stages of the affection. Their skin has a great tendency to assume that appearance which has been denominated 'goose skin ;' and if the weather be cold, small brown or dark spots will be observed at the roots of the hairs. These symptoms would soon terminate in evident scorbutic disease if the cause would continue in operation. But it would seem, that at this stage of the affection the natural instinct of every man, who is a free agent, becomes so urgent in prompting him to endeavour to procure such aliment as he has been deficient of, and which is of course the very aliment which nature at this moment requires for repair of waste and support ; that nothing except the most urgent necessity can oppose him in obtaining it. Thus the affection of the constitution is prevented from getting worse, and may be in the same manner prevented for years ; and the appearance of prominent scorbutic symptoms are also prevented, but the habit continues sufficiently depraved to favor the attack of any disease that may happen to be prevalent or epidemic ; and to enable that disease, whatever its nature may be, to become much more malignant and fatal." 37.

Mr. Cameron justly remarks that instinct has a much more extensive influence on our habits and feelings than our vanity, assisted by erroneous ideas, will allow us to admit.

"We see daily examples, where, if a person has continued for some time to live on any particular species of aliment, he will gradually begin to loathe this diet, and experience a craving for some other article of food. Here we mark the force and use of instinct. The elements of the food on which the individual has been living not containing all the ingredients required for the nourishment and health of his frame, vitality finds out this secret, and taking the alarm before much injury is done, communicates the important information, in some mysterious manner, to the stomach; which, after the receipt of such intelligence, refuses, as far as possible, the former aliment, and loudly demands a change of diet." 37.

Mr. Cameron conceives that, from the structure of the teeth and digestiye organs, it is evident that "man was not intended to be carnivorous, *except in a slight degree*, and only to a limited extent." The author should recollect the case of the South Americans, who live almost exclusively on meat, and the Irish, who feed almost entirely on vegetables, yet both continue in good health—at least in as good health as John Bull, who is a truly omnivorous animal.

Of Mr. Cameron's disputation with Dr. Stevens we shall take no notice.

"That cholera makes its attacks only on persons affected with the depraved habit of body under consideration, will not admit of any doubt; indeed the affection seems to be incompatible with any other state of the constitution. The doctrine of contagion in this disease is as idle and silly as any legend ever invented by the elders of catholicism, to impose the belief of the perfection of their saints on a weak-minded, ignorant, and credulous populace. It is also a mistake to suppose that the disease is new to this country. That cholera is now more frequently met with than at any former period is evident, but it has been at all periods and ages common enough in England. I have seen it here years ago, as strongly characterised as I ever saw it in India, even in 1817-18. The only case of cholera which has hitherto proved fatal under my care was at Woolwich, in March 1826. The patient had incessant vomiting and purging, spasms, total suppression of urine, and livid colour of the skin all over the body. He died in about ten hours after I saw him, and about twenty-three hours after the attack commenced. When I began the treatment of this case, I had reason to fear that venesection was too late. I knew the disease well. I had seen a great deal of it in India previous to this period." 97.

Mr. Cameron concludes his volume thus:—

"Let man, therefore, return to the diet which Nature intended for him, and which is evidently much better adapted to his wants; let him use a much smaller proportion of animal food, and a much larger of the farinaceous grains, as preparations of wheat, oats, barley, rye, &c. Let milk form part of his aliment and let him add to this as much of the common vegetables, and fruits in season, as his instinct will prompt him to. Let him avoid spirituous liquors except as a cordial. Let him do all this, and he will enjoy far better health than he does at present. All epidemic, pestilential, malignant, and putrid diseases, will disappear: and many now considered as contagious or infectious, will be no longer known on the face of the earth." 104.

It is not our intention to make many comments on Mr. C.'s book. Few will disagree with him, that it is desirable as well as pleasant to vary the quality of our food, and to range through the animal and vegetable kingdom in search of variety. It is also very probable that we eat much more animal food than is necessary or proper,—and, consequently, that, in civilised life, we predispose the constitution to many inflammatory and other complaints; but that any plan of diet which Mr. Cameron could devise, would cause epidemic diseases to disappear, is more than we can believe. We wish Mr. Cameron may realize his anticipations, and continue to be as successful in practice as he appears hitherto to have been.

